# Resting alpha activity predicts learning ability in alpha neurofeedback

**DOI:** 10.3389/fnhum.2014.00500

**Published:** 2014-07-14

**Authors:** Feng Wan, Wenya Nan, Mang I. Vai, Agostinho Rosa

**Affiliations:** ^1^Department of Electrical and Computer Engineering, Faculty of Science and Technology, University of MacauTaipa, Macau; ^2^Department of Bio Engineering, Systems and Robotics Institute, Technical University of LisbonLisbon, Portugal

**Keywords:** neurofeedback, prediction, learning ability, alpha band, resting baseline

## Abstract

Individuals differ in their ability to learn how to regulate the brain activity by neurofeedback. This study aimed to investigate whether the resting alpha activity can predict the learning ability in alpha neurofeedback. A total of 25 subjects performed 20 sessions of individualized alpha neurofeedback and the learning ability was assessed by three indices respectively: the training parameter changes between two periods, within a short period and across the whole training time. It was found that the resting alpha amplitude measured before training had significant positive correlations with all learning indices and could be used as a predictor for the learning ability prediction. This finding would help the researchers in not only predicting the training efficacy in individuals but also gaining further insight into the mechanisms of alpha neurofeedback.

## Introduction

Neurofeedback is devoted to training people to gain control over the electro-physiological processes in the human brain. An increasing number of studies have demonstrated that neurofeedback is a potential and non-pharmacological supportive treatment for many neurological and psychiatric disorders which are accompanied by abnormal patterns of cortical activity such as attention deficit hyperactivity disorder (ADHD; Arns et al., 2009; Duric et al., 2012; Moriyama et al., 2012), depression (Choi et al., 2011; Dias and van Deusen, 2011), substance abuse (Sokhadze et al., 2008), and schizophrenia (Bolea, 2010; Nan et al., 2012a; Surmeli et al., 2012). In addition to clinical applications, neurofeedback has also shown the potential for skills enhancement in healthy individuals (Vernon, 2005; Gruzelier et al., 2006; Gruzelier, 2013a), e.g., cognitive abilities (Vernon et al., 2003; Hanslmayr et al., 2005; Zoefel et al., 2011), memory (Nan et al., 2012b; Wang and Hsieh, 2013), peripheral visual performance (Nan et al., 2013), creativity and artistic performance (Egner and Gruzelier, 2003; Gruzelier, 2009, 2013b; Gruzelier et al., 2010).

Although neurofeedback has demonstrated benefits in many aspects, not all subjects have shown satisfactory learning ability to regulate brain activity. Prior research classified the subjects into learners or non-learners according to their learning ability. Some studies reported the cases of non-learners even after repeated training sessions (Kotchoubey et al., 1999; Hanslmayr et al., 2005; Kropotov et al., 2005; Doehnert et al., 2008; deBeus and Kaiser, 2011; Escolano et al., 2011; Weber et al., 2011; Zoefel et al., 2011; Enriquez-Geppert et al., 2013b; Kouijzer et al., 2013). In Weber et al. (2011), about 50% of subjects were non-learners in sensorimotor rhythm (SMR; 12–15 Hz) neurofeedback. Among others, the studies in Enriquez-Geppert et al. (2013b) for frontal-midline theta neurofeedback, in Zoefel et al. (2011) and in Hanslmayr et al. (2005) for upper alpha neurofeedback, reported 25%, 21.4%, and 50% of the subjects found to be non-learners, respectively.

The learning ability in neurofeedback is important since it has a crucial mediation link with neurofeedback training outcome (Gruzelier, 2013a). For example, the musical performance improvement had high correlation with learning to progressively raise theta over alpha band amplitudes (Egner and Gruzelier, 2003). Some studies have further demonstrated that only the individuals who successfully learn to self-regulate the brain activity can achieve behavior improvement. Kouijzer et al. (2013) reported neurofeedback training for autism spectrum disorders, in which only the participants who significantly reduced their delta and/or theta power during neurofeedback sessions showed significant improvement in cognitive flexibility. Similarly, in Hanslmayr et al. (2005), only the learners showed enhanced performance in a mental rotation task after upper alpha neurofeedback training.

An interesting question in neurofeedback research is that the assessment of learning ability varies among studies. Some researchers defined the learner by the training parameter changes between two periods, e.g., between the last session and the baseline (Zoefel et al., 2011), or between the first session and the last session (Dekker et al., 2014). The learning ability was also identified by the changes across the whole training course, e.g., a learner was referred to the subject who showed a significant correlation between the training parameter during session and the session number (Kouijzer et al., 2013). However, Dempster and Vernon (2009) suggested that within session changes may be more useful to identify changes resulting from neurofeedback. Additionally, some studies adopted more than one of the above assessment methods. For instance, Enriquez-Geppert et al. (2013a) employed two different learning indices: one was the training parameter changes within sessions and another was the training parameter changes across the whole training course. A different example is from Weber et al. (2011), in which a learner should meet two criteria simultaneously, i.e., Criterion 1: the mean percentage increase in the training frequency band in the last five training days exceeded 8% of the baseline, and Criterion 2: the mean amplitude change across all sessions was positive. In summary, the assessment of learning ability is mainly from three aspects, i.e., changes between two periods, changes within a short period and changes across the whole training time. In our opinion, the assessment criterion should be related to the researcher’s training objective. If the researcher aims to investigate the accumulative training effect, the learning ability can be assessed by the changes across the whole training time. If the researcher aims to investigate how the training parameter changes, the learning ability can be assessed by the changes within a short period, across the whole training time, or between two periods. Nevertheless, if the training objective is to enhance a performance, the learning ability should be assessed by the changes related to the performance.

Apart from the learning ability assessment, another important question is, whether there are any parameters related to or factors affecting the learning ability. For the frontal-midline theta neurofeedback, the learning ability does not result from the motivation or commitment (Enriquez-Geppert et al., 2013b) but can be predicted by the volume of the mid cingulate cortex as well as the volume and concentration of the underlying white matter structures of the subjects (Enriquez-Geppert et al., 2013a). Another research group tried to find out the parameters related to the learning ability of SMR neurofeedback from the psychological aspect (Kober et al., 2013; Witte et al., 2013). It was found that control beliefs and mental strategies affected the training result of SMR neurofeedback, while mental strategies could not affect the training result of gamma neurofeedback (Kober et al., 2013). With respect to slow cortical potential (SCP) neurofeedback, the initial performance level has been shown to have some predictive value in SCP neurofeedback response (Kotchoubey et al., 1999; Neumann and Birbaumer, 2003). In a word, the parameters related to or the factors affecting the learning ability are different among different neurofeedback paradigms, depending upon some psychological and physiological mechanisms.

Regarding alpha neurofeedback, positive effects have been shown on cognition and memory enhancement as well as clinical treatment (Hanslmayr et al., 2005; Escolano et al., 2011; Zoefel et al., 2011; Nan et al., 2012b; Hartmann et al., 2014). However, the learning ability also shows inter-individual difference (Hanslmayr et al., 2005; Escolano et al., 2011; Zoefel et al., 2011). Nan et al. (2012b) investigated whether mental strategy had an effect on the training performance of alpha neurofeedback. The participants were required to write down and score their mental strategy. It was found that most participants utilized positive strategies during training and the efficient strategies varied among individuals. On average, the most successful mental strategies were related to positive strategies, namely friends, love and family.

Besides mental strategy, whether physiological parameters (e.g., pre-training EEG) can predict the learning ability in alpha neurofeedback is unknown. The development of predictors based on pre-training EEG would help the researcher in not only predicting the training efficacy in individuals but also gaining further insight into the mechanisms of alpha neurofeedback.

Thus, this study aimed to investigate whether the resting alpha activity measured before training was correlated to the learning ability in alpha neurofeedback and could be used as a predictor. More specifically, we assessed the learning ability with all the aforementioned assessment methods respectively: the training parameter changes between two periods, within a short period and across the whole training time. Due to the large inter-individual differences in the alpha frequency band (Klimesch, 1999; Klimesch et al., 2003), we utilized the individual alpha band rather than the standard alpha band (8–12 Hz) for neurofeedback training.

## Method

### Subjects

A total of 25 healthy subjects (16 males) participated in the neurofeedback training. The mean age of the subjects was 23.12 years (standard deviation (SD) 3.31, range 18–33). Inclusion criteria for the neurofeedback were as follows: no history of psychiatric or neurological disorders, no psychotropic medications or addiction drugs, and with normal or corrected-to normal vision. Prior to the experiment, informed written consent was obtained from all subjects after the experimental nature and procedure were interpreted to them. The protocol was in accordance with the Declaration of Helsinki and approved by the Research Ethics Committee (University of Macau).

### Neurofeedback training

Each subject completed neurofeedback training with 3 or 4 sessions per day for a total of 20 sessions. Each session was composed by 10 successive trials of 20 s each and with an interval of 5 s between two consecutive trials. Before and after all neurofeedback sessions, two 30-s epochs with eyes open and two 30-s epochs with eyes closed resting baseline were recorded.

EEG signal was acquired at Cz according to the International 10–20 system. The reference electrodes were placed on the left and right mastoids, and the ground was located at the forehead. The signals were amplified by an EEG amplifier (Vertex 823 from Meditron Electomedicina Ltda, SP, Brazil) with an analog band-pass filter from 0.1 to 70 Hz and recorded by a Somnium system (Cognitron, SP, Brazil) at a sampling frequency of 256 Hz. In the Somnium system, the signals were filtered by a band-pass filter from 0.5 to 30 Hz, and a notch filter at 50 Hz. The impedance was maintained below 10 kΩ for all electrodes.

**Figure 1:**
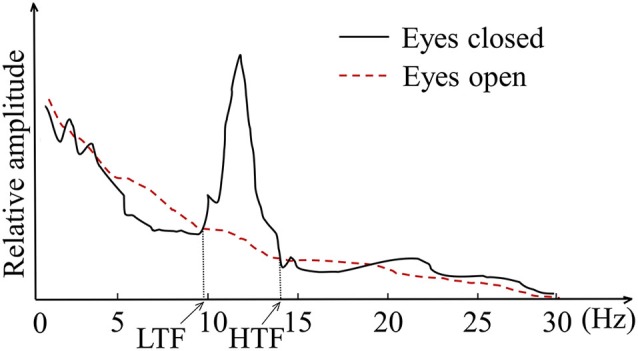
**The illustration of individual alpha frequency band**.

The subjects were trained in the individual alpha band which was determined by the crossings between the eyes-open resting baseline and the eyes-closed resting baseline measured before training (Klimesch, 1999). As demonstrated in Figure 1, the individual alpha band ranged from the low transition frequency (LTF) to the high transition frequency (HTF). The training parameter was the relative amplitude in the individual alpha band, or the (relative) alpha amplitude for short, which can be calculated using Equation (1), where *X*(*k*) was the frequency amplitude spectrum calculated by fast Fourier transformation (FFT) with a sliding window of 2 s that shifted every 0.125 s. Thus the frequency resolution was 0.5 Hz.

(1) $$\text{relative alpha amplitude}=\frac{\frac{\sum_{k=LTF}^{HTF}X\left(k\right)}{HTF-LTF}}{\frac{\sum_{k=0.5}^{30}X\left(k\right)}{30-0.5}}$$

**Figure 2:**
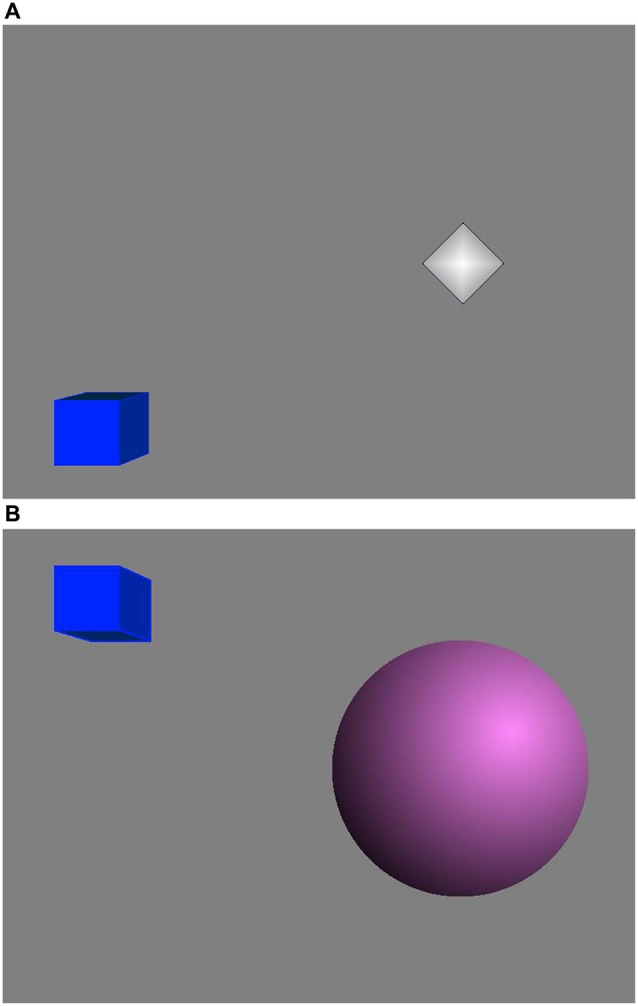
**Neurofeedback display**. **(A)** When Goal 1 was not achieved. **(B)** When both Goal 1 and Goal 2 were achieved.

The feedback display contained two 3D objects: a sphere and a cube (Figure 2). The sphere radius reflected the feedback parameter in real time and if this value reached a threshold (Goal 1) the sphere color changed. This sphere was made of several slices and the more slices it had, the smoother it looked. The cube height was related to the period of time for which Goal 1 kept being achieved continuously. If Goal 1 was being achieved continuously for more than a predefined period of time (2 s), Goal 2 was accomplished and the cube rose up until Goal 1 stopped being achieved. Then the cube started falling slowly until it reached the bottom or Goal 2 was achieved again (Rodrigues et al., 2010; Nan et al., 2012b). Therefore, the subjects were instructed to apply mental strategies to increase the sphere size or keep the cube as high as possible. No instructions about the effective mental strategies were given since the effective mental strategies vary among individuals (Nan et al., 2012b).

The feedback threshold was set to 1 in the first session, and it could be adjusted according to the session report which showed the percentage of time for alpha above threshold in each session. If the percentage of time exceeded 60%, the threshold would be increased by 0.1 in the next session. In contrast, if this value was below 20%, the threshold would be decreased by 0.1 afterward.

### Learning indices

In this study, the learning ability was assessed from three different aspects, i.e., the training parameter changes between two periods, within a short period and across the whole training time. These three aspects covered the assessment methods in current neurofeedback research as described in Introduction section.

For the training parameter changes between two periods, the first learning index (L1) was the difference between Session 1 and Session 20 since the short term memory enhancement was found significantly correlated with the increase from the first training session to the last training session (Nan et al., 2012b).

For the training parameter changes within a short period, the period was chosen as one training day. The total training time in each training day was 10 min or 13.4 min since each training day consisted of 3 or 4 sessions and each session had 10 20-s trials and 5-s interval between two consecutive trials. We firstly calculated the training parameter changes of each session relative to the first session of the corresponding training day as the within-day change. And then the mean within-day change across all training days was taken as the second learning index L2 shown in Equation 2.

(2) $$\text{L2}=\frac{\sum_{i=1}^{\text{total training day}}\sum_{j=2}^{\text{total session in }i\text{-th training day}}\left(\text{session }j\text{-session 1 of }i\text{-th training day}\right)}{\text{total training day}}$$

Regarding the changes across the whole training time, we focused on the alpha amplitude over sessions. Considering the nonlinear trend of alpha over sessions, the third learning index (L3) was the slope of the regression line calculated by a logarithmic regression model in which the session number was taken as the independent variable and the alpha amplitude during sessions was the dependent variable, indicating the learning speed across the whole training time.

### Data analyses

The mean alpha amplitude in each session and the resting baseline measured before training was computed, and all learning indices were calculated for each subject. SPSS Statistics 20 (SPSS, Chicago, USA) was used and the significance level was set as *p* < 0.05 for the following statistical analyses. Data distribution was analyzed by one-sample Kolmogorov-Smirnov test. All data were found normally distributed except for L2. By further investigation, one subject’s L2 was recognized as outlier and resulted in non-normal distribution. In order to make fair comparison between all learning indices, we excluded this subject in the subsequent analyses.

Initially the mean, range, and SD were calculated for all learning indices and the resting alpha feature before training (amplitude, HTF, LTF). The eyes-open and eyes-closed resting alpha amplitudes before training were compared by paired *t*-test. In order to examine the correlation between each learning index and the alpha amplitude in both eyes-closed and eyes-open resting condition, 2-tailed Pearson correlation test was applied. Moreover, for each learning index (L1, L2, L3), two linear regression analyses were set up. One regression analysis used the eyes-open resting alpha amplitude as the predictor variable, and the other used the eyes-closed resting alpha amplitude.

Besides, we also examined the alpha amplitude changes over the course of training by repeated-measures analysis of variance (ANOVA). Here the amplitude change of each session was quantified as the change relative to the first training session according to Enriquez-Geppert et al. (2013a). In addition, the correlation of the alpha amplitude changes between the second session and the last session was examined by one-tailed Pearson correlation test since prior studies reported a significant correlation between the early training effects and the final training outcome (Neumann and Birbaumer, 2003; Weber et al., 2011; Enriquez-Geppert et al., 2013a).

## Results

Regarding the resting alpha feature measured before training, the LTF ranged from 6.2 Hz to 10 Hz (mean: 7.89 Hz, SD: 0.89 Hz) and the HTF was between 10.2 Hz and 13.3 Hz (mean: 11.93 Hz, SD: 0.69 Hz). Moreover, the alpha amplitude varied between 0.9 and 3.15 (mean: 1.83, SD: 0.59) in the eyes-closed resting condition, while in the eyes-open resting condition it was in the range of 0.73 to 1.93 (mean: 1.09, SD: 0.25). Paired *t*-test revealed a significant difference in the alpha amplitude between the eyes-open and eyes-closed resting condition (*t*_(23)_ = 8.334, *p* < 0.001). For the learning indices, L1 ranged from −0.26 to 0.63 (mean: 0.18, SD: 0.22), L2 ranged from −0.15 to 0.57 (mean: 0.14, SD: 0.19), and L3 ranged from −0.053 to 0.224 (mean: 0.062, SD: 0.065).

**Figure 3:**
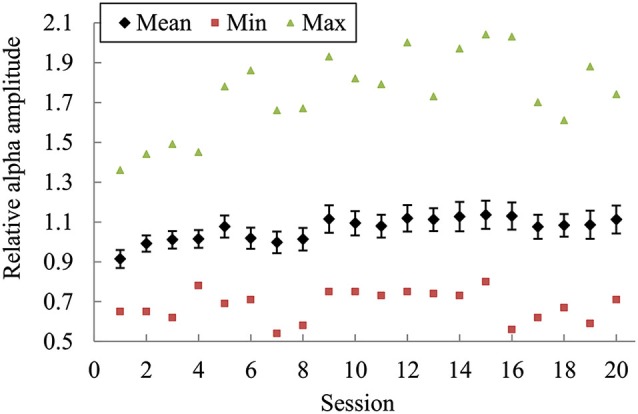
**The mean (± its standard error) of alpha amplitude across all subjects as well as the minimum and maximum values in each training session.** The bars indicate the standard error of the mean.

Figure 3 presents the mean (± its standard error) of alpha amplitude across all subjects as well as the minimum and maximum values in each training session. On average, the alpha amplitude showed an increasing trend across the whole training process. Repeated-measures ANOVA revealed a significant main effect of session on the alpha amplitude changes over the course of training [*F*_(18, 437)_ = 3.333, *p* < 0.001, partial *η*^2^ = 0.127]. Further pairwise comparisons found the alpha amplitude changes in Session 5 and Sessions 9 to 20 significantly higher compared with the changes in Sessions 2 to 4. However, no significant correlation was found between the changes in the second session and in the last session.

Table 1 shows the correlation test results between the learning indices and the resting alpha amplitude. As depicted in this table, all indices had significant positive correlations with the resting alpha amplitude during the eyes-open and eyes-closed conditions. Furthermore, the resting alpha amplitude during the eyes-closed condition had higher correlation coefficients with all learning indices than the eyes-open condition.

**Table 1:** **Pearson correlation results between resting alpha amplitude and learning indices**.

Condition	L1	L2	L3
Eyes-open	*r* = 0.456 (*p* < 0.05)	*r* = 0.432(*p* < 0.05)	*r* = 0.540 (*p* < 0.01)
Eyes-closed	*r* = 0.470 (*p* < 0.05)	*r* = 0.547 (*p* < 0.05)	*r* = 0.631 (*p* < 0.01)

**Figure 4:**
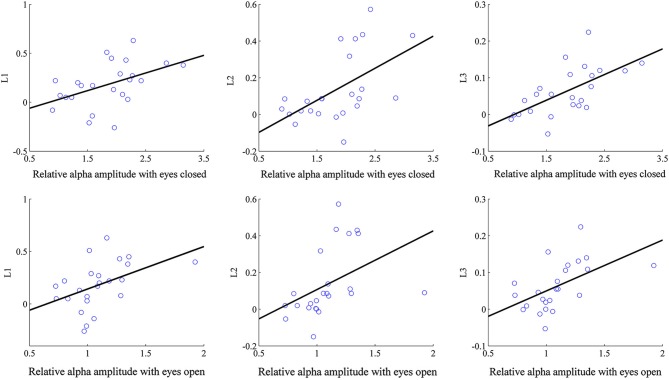
**Correlation of the eyes-closed/eyes-open resting alpha amplitude with the learning indices**. Each circle corresponds to one subject.

We applied linear regression analyses with the eyes-open resting alpha and eyes-closed resting alpha as predictors respectively. When the eyes-open resting alpha amplitude was selected as the predictor, linear regression model *R*^2^ was 0.208 for L1 (*p* = 0.025), 0.186 for L2 (*p* = 0.035), and 0.291 for L3 (*p* = 0.007). Therefore the eyes-open resting alpha amplitude was identified as a significant predictor which accounted for 20.8% of the variance in L1, 18.6% of the variance in L2, and 29.1% of the variance in L3. When the eye-closed resting alpha amplitude was taken as the predictor, it accounted for 22.1% of the variance in L1 (*p* = 0.020), 29.9% of the variance in L2 (*p* = 0.006), and 39.8% of the variance in L3 (*p* = 0.001). By comparisons of the above regression results, it can be observed that the resting alpha amplitude in the eyes-closed condition explained higher variance than that in the eyes-open condition for all learning indices. Moreover, the eyes-closed resting alpha provided the best prediction in L3. In Figure 4, the eyes-closed/eyes-open resting alpha amplitudes are plotted against the learning indices in alpha neurofeedback, and each solid line results from the linear regression analysis of the corresponding learning index onto its predictor.

## Discussion

Neurofeedback training has been shown benefits on human cognition, health and task performance. However, individuals differ in their learning ability in neurofeedback which has the crucial mediation link with neurofeedback training outcome (Gruzelier, 2013a). This study aimed to investigate whether the resting alpha activity measured before training was correlated to the learning ability of alpha neurofeedback and could be used as a predictor. The learning ability was assessed from three different aspects including the changes between two periods, within a short period and across the whole training time. It was found that the resting alpha amplitude during the eyes-open or eyes-closed condition measured before training was significantly correlated with all learning indices. What’s more, the resting alpha amplitude was a predictor for the learning ability. Our results suggested that the learning ability can be predicted before neurofeedback training.

The average range of the individual alpha band of the subjects in this study was 7.89 to 11.93 Hz, with no significant difference from the standard (8–12 Hz). However, the measurement of the individual alpha band showed apparent inter-individual difference. This indicates the importance of adapting the feedback to the individual alpha band. Moreover, inter-individual difference was observed in the alpha amplitude during the resting condition, which is reasonable since the alpha amplitude can be influenced by a range of anatomical and functional factors including tissue conductivity, cerebral blood flow, hormonal and neurohumoral factors, electromyogenic artifacts, etc (Bazanova and Vernon, 2013).

Regarding the EEG during training, the mean alpha amplitude across all subjects showed an increasing trend over time, which is in agreement with previous research showing alpha enhancement over the course of training (Dempster and Vernon, 2009; Zoefel et al., 2011; Dekker et al., 2014). On the other hand, the results in this study also indicated a main effect of session on the alpha amplitude changes. This is in line with Enriquez-Geppert et al. (2013a) in which the main effect of the session in the repeated-measures ANOVA testing was significant for the trained theta amplitude changes over the course of training. However, different from prior neurofeedback studies (Neumann and Birbaumer, 2003; Weber et al., 2011; Enriquez-Geppert et al., 2013a), we failed to find a significant correlation of the alpha amplitude changes between the second and the last session. This may be due to the small sample size. Another possible explanation is that the neurofeedback protocols are different across studies, including the training schedule, session duration, and session number.

Apart from overall enhancement across all subjects, we also found the inter-individual difference on alpha enhancement. In particular, the difference between the first session and the last session (i.e., L1) is positively correlated with the short term memory enhancement (Nan et al., 2012b). The present work found that L1 can be predicted by the resting alpha amplitude before training, indicating that the enhancement in short term memory may also be predicted by the same predictor. Besides L1, the learning ability was also assessed by the changes within-day (L2) and across the whole training time (L3). All learning indices had significant positive correlations with the resting alpha amplitude, and they can be predicted by the resting alpha amplitude measured before training. Particularly, the eyes-closed resting alpha provided the better prediction than the eyes-open resting alpha, and L3 got the best prediction compared to L1 and L2.

Prior neurofeedback studies tried to predict the learning ability in other training paradigms from different perspectives. For instance, Enriquez-Geppert et al. (2013a) investigated the prediction of the frontal-midline theta neurofeedback training success from the brain structures. It was reported that the volume of the mid cingulate cortex as well as the volume and concentration of the underlying white matter structures acted as predictor variables for the general responsiveness to training. Additionally, Weber et al. (2011) found that the achieved augmentation of SMR with a total of 25 sessions could be predicted based on the outcome of the first 11 sessions. The present study found that the prediction of the learning ability to regulate alpha activity can be done based on the resting alpha amplitude before training. Similarly, in brain computer interfaces (BCIs), some studies reported that BCI performance can be predicted by the eyes-open or eyes-closed resting EEG feature before BCI task (Blankertz et al., 2010; Treder et al., 2011). For instance, BCI performance in a motor imagery paradigm can be predicted by a predictor which was determined from a 2-min recording of a relaxation with eyes-open condition using two Laplacian EEG channels (Blankertz et al., 2010). The present study also found that the eyes-open or eyes-closed resting alpha could be used as the predictor of the learning ability in alpha neurofeedback, and the highest correlation coefficient was between the eyes-closed resting alpha amplitude and L3 (*r* = 0.631).

In a practical viewpoint, the advantages of the finding in this study are economical, convenient and time saving as the prediction only needs a few minutes of resting EEG recording from one channel before training. On the other hand, this finding may help researchers to understand more about the alpha neurofeedback mechanism. Neurofeedback may be producing effects by enhancing circuitry and modulating the brain networks including the default mode network (DMN), the central executive network (CEN) and the salience network (SN; Niv, 2013). In particular, neurofeedback regulating the DMN activity improves the brain’s self-regulation capabilities (Othmer et al., 2011), and the alpha reduction neurofeedback increases the connectivity within the SN regions involved in intrinsic alertness (Ros et al., 2013), whereas alpha enhancement neurofeedback leads to higher outgoing connectivity in a neighboring region of the training area (Hartmann et al., 2014). Furthermore, contemporary neuroscience has regarded alpha enhancement as an active top-down inhibitory process for the exclusion of conflicting or irrelevant inputs (Gruzelier, 2013a). A greater level of alpha amplitude reflects the inhibition of non-essential activity which in turn may facilitate performance on the task (Klimesch et al., 2007). Therefore, higher resting alpha amplitude may lead to stronger inhibiting irrelevant processes during neurofeedback so that it is associated with higher learning ability to regulate the alpha amplitude during training.

In conclusion, the alpha amplitude during the eyes-open or eyes-closed resting condition could predict the learning ability in alpha neurofeedback training. This finding would help us in predicting the training efficacy in individuals and also provide new insights about the mechanisms of alpha neurofeedback for further improvement of the training effectiveness.

## Conflict of interest statement

The authors declare that the research was conducted in the absence of any commercial or financial relationships that could be construed as a potential conflict of interest.
